# Time perception impairs sensory-motor integration in Parkinson’s disease

**DOI:** 10.1186/1755-7682-6-39

**Published:** 2013-10-16

**Authors:** Marina Lucas, Fernanda Chaves, Silmar Teixeira, Diana Carvalho, Caroline Peressutti, Juliana Bittencourt, Bruna Velasques, Manuel Menéndez-González, Mauricio Cagy, Roberto Piedade, Antonio Egidio Nardi, Sergio Machado, Pedro Ribeiro, Oscar Arias-Carrión

**Affiliations:** 1 Brain Mapping and Sensory Motor Integration, Institute of Psychiatry of Federal University of Rio de Janeiro (IPUB/UFRJ), Rio de Janeiro, Brazil; 2 School of Physical Education, Bioscience Department (EEFD/UFRJ), Rio de Janeiro, Brazil; 3 Division of Epidemiology and Biostatistic, Institute of Health Community, Federal Fluminense University (UFF), Rio de Janeiro, Brazil; 4 Institute of Applied Neuroscience (INA), Rio de Janeiro, Brazil; 5 Physiotherapy Laboratory, Veiga de Almeida University (UVA), Rio de Janeiro, Brazil; 6 Neurology Unit, Hospital Álvarez-Buylla, Mieres, Spain; 7 Laboratory of Panic and Respiration, Institute of Psychiatry, Federal University of Rio de Janeiro (IPUB/UFRJ), Rio de Janeiro, Brazil; 8 National Institute of Translational Medicine (INCT-TM), Rio de Janeiro, Brazil; 9 Faculty of Medical Sciences, Quiropraxia Program, Central University, Santiago, Chile; 10 Physical Activity Neuroscience, Physical Activity Postgraduate Program, Salgado de Oliveira University (UNIVERSO), Niterói, Brazil; 11 Sleep and Movement Disorders Clinic and Transcranial Magnetic Stimulation Unit, Hospital General Dr. Manuel Gea González, México D.F., Mexico; 12 Sleep and Movement Disorders Clinic and Transcranial Magnetic Stimulation Unit, Hospital General Ajusco Medio, México D.F., Mexico

**Keywords:** Parkinson’s Disease, Sensorimotor integration, Time perception

## Abstract

It is well known that perception and estimation of time are fundamental for the relationship between humans and their environment. However, this temporal information processing is inefficient in patients with Parkinson’ disease (PD), resulting in temporal judgment deficits. In general, the pathophysiology of PD has been described as a dysfunction in the basal ganglia, which is a multisensory integration station. Thus, a deficit in the sensorimotor integration process could explain many of the Parkinson symptoms, such as changes in time perception. This physiological distortion may be better understood if we analyze the neurobiological model of interval timing, expressed within the conceptual framework of a traditional information-processing model called “Scalar Expectancy Theory”. Therefore, in this review we discuss the pathophysiology and sensorimotor integration process in PD, the theories and neural basic mechanisms involved in temporal processing, and the main clinical findings about the impact of time perception in PD.

## Introduction

When individuals observe a slow-motion movement, the central nervous system (CNS) speeds up or reduces the time in order to balance the movement feedback and visual perception [1]. In other words, when an individual watches a movie scene in slow-motion for a few seconds, the CNS reorganizes the time estimation according to the normal speed of movement; this leads to the impression of shortening of time [2,3]. This temporal information processing is deficient in PD patients and the range of milliseconds seems to be impaired; this results in temporal judgment deficits [4,5].

Several areas of the cerebral cortex have been associated with time perception (TP), such as prefrontal cortex (PFC), right postcentral gyrus, inferior parietal lobe (IPL), basal ganglia (BD) and cerebellum [6]. In addition to these areas, premotor cortexes (PMC), supplementary motor areas (SMA) and anterior cingulate cortexes (aCC) seem to be more active during situations in which the individual needs to discriminate between two events at different times [7,8]. Other experiments have shown that impairment of the posterior parietal cortex (PPC) and basal ganglia (BG) decreases the time perception between the stimuli recognition and the utilization of the right stimulus in different occasions [9,10].

The set of PD symptoms generates a series of physical, psychological and intellectual impairments that compromise the ability to perform daily living activities such as making temporal judgments [11-14]. Furthermore, the neural networks of the human internal clock and temporal memory suffer changes in PD [15,16]. Thus, our review aims to present the Pathophysiology, sensorimotor integration process in PD, the theories and neural basic mechanisms involved in TP, and the main clinical findings about the impact of PD on TP.

### Basic mechanisms involved in time perception

The way the human brain encodes and organizes information to generate a temporal response is still poorly understood [17]. Current theories support the hypothesis that human time perception is regulated by a single internal clock which can be switched off or paused [18]. However, the relativity theory indicates that time depends on different factors and its perception is recorded independently by different internal clocks, affected by intrinsic and psychological mechanisms. Considering this, researchers formulated two hypotheses: the first one states that time is independent from internal clocks; the second hypothesis supports the idea that internal clocks use different temporal contexts to judge the duration of a certain event [16,19,20].

Therefore, it seems that temporal processing requires the mutual participation of three neural systems; the first one consists of a “core timer”, the second one is an executive system that processes the stimulus duration, and the third one is an associative system for contextualizing the duration of the stimuli [21,22]. On the other hand, Gibbon et al. [23] presented the Scalar Expectancy Theory, widely used in current research as one of the most accepted models of time processing. This theory consists of an intrinsic pacemaker responsible for producing pulses to estimate the length of time. The CNS compares the different information about time and stores it inside an accumulator that counts the pulses from the pacemaker. If the duration of the signal received by the accumulator becomes meaningful, the stored content is transferred from working to long-term memory [18,24,25].

Another TP theory argues that two parallel systems operate in a temporal integration mechanism [26]; the first system, or “bottom up”, is responsible for synchronizing milliseconds, which are important for motor coordination (cerebellum), the second system, or “top down”, synchronizes seconds and minutes (frontostriatal circuits) [27]. Other experiments show that the CNS first processes time discrimination and judgment and after, it controls time and rhythm perception [28]. In this context, studies have shown activation in certain cortical areas, such as the thalamus, the substantia nigra, striatum, SMA and the prefrontal cortex, during time perception and integration. In particular, the striatum acts as the core timer, controlling the time networks [22]. Studies in rats have shown that the ability to measure the duration of a previously taught task is lost when striatal injury occurs [23,29].

The ability to focus on a particular event, as well as the intuition to anticipate occurrences can promote changes in TP. Studies show the existence of two factors that influence the subjective perception of time. The first factor is the focus, which can accumulate the number of nervous pulses per in a time unit. The second one is the level of arousal, which can increase the number of pulses emitted per time unit [16,30]. As an example, consider the group of sequential numbers (1, 2, 3, 4); the first number is processed with a longer perception than the following one, but when the numbers are shuffled (2, 4, 3, 1), they are all processed with the same duration; i.e., neural networks do not have to anticipate the order, and they have the same degree of neural processing [31]. This fact demonstrates that events requiring more attention and more neural processing lead to longer time perception [32-34].

The variation of our sensations depends on several time mechanisms. When a given temporal or non-temporal event captures our attention, processing mechanisms may go to the various time controllers, causing a decrease in the pulses of the pacemaker. Consequently, there is an underestimation of time, i.e. the individual has the perception of a time shorter than the actual one [35]. For example, the underestimation of time frequently occurs as we remember an enjoyable activity, when time seems to go faster. This happens because we pay less attention to the passing of time due to the fact that the attention is being directed to another activity; this produces a smaller number of pulses counted by the accumulator, and leads to the false impression that time passed quickly [16,36]. Moreover, time may be overrated when we experience a subjective sensation of prolonged time. This occurs when there is an increase in excitation or in attention to time and this causes an acceleration of the pacemaker, which accumulates a greater number of pulses proportionally to the physical time [37].

### Pathophysiological mechanisms of PD

Approximately four million people worldwide are affected by Parkinson’s Disease. An increase in the incidence of 1.4% in individuals aged 65–75 and 4.3% in individuals aged over 85 was estimated [38]. In addition, there are reports that family members of PD patients present some olfactory and cognitive deficits. This indicates that such deficits could be a pre-clinical stage of Parkinson’s Disease [39,40]. Though, other studies show that such a relationship does not exist, and argue that occurrence is due to exposure to the same unknown environment [40,41]. However, there are two genetic factors related to cognitive dysfunction in PD: the genes for catechol-O-methyltransferase and microtubule associated tau protein [42].

In general, the pathophysiology of PD has been described as a dysfunction in the BG circuits responsible for failures in cortical and subcortical neural networks [43-47]. Meanwhile, Parkinson’s syndrome can be triggered by a variety of conditions such as exposure to dopamine-blocking agents, exporure to toxins, traumatic brain injury, cerebral infarction, encephalitis, metabolic disorders, among others [48]. In both cases, the BG play an important role in cognitive, executive and movement planning functions [49,50]. They are essential for facilitating voluntary movements and for inhibiting unwanted ones. Therefore, disturbances in BG mechanisms impair all types of motor control [51].

Among the many different causes of impairment in the basal ganglia, the decrease in dopamine release by the substantia nigra to the striatum (putamen) appears to explain movement disorders in PD [9,37,38,52-59]. Two important circuits in the basal ganglia are affected by reduced activity of dopamine-secreting cells in the substantia nigra. The first one is a direct circuit responsible for triggering the movement and for activating the neurons in the thalamocortical route. The second one is an indirect circuit that inhibits the neurons in the path from the thalamus to the cortex [60]. The impairment of these circuits is related to many of the Parkinson symptoms, such as changes in time perception [61]. During disease progression, some exacerbate symptoms and complications develop, such as: falls, postural instability, gait freezing, postural hypotension and severe dementia. Concurrent with the emergence of these events, there is a gradual loss of cognitive ability and use of language, reasoning and memory [62,63].

### First movement alterations in people with Parkinson's disease

Bradykinesia was explained initially by James Parkinson, as one of the major clinical symptoms in patients with PD [64]. This term, also called akinesia or hypokinesia, characterizes the difficulty of planning, executing or starting motion and interferes especially with fine motor daily living activities in PD [65,66]. There are some factors that contribute to this phenomenon, such as muscle weakness, stiffness, tremor, thought slowness and motion instability [67]. The cause of muscle weakness still leaves open questions; however, research with electromyography (EMG) in PD showed that the trigger of a voluntary contraction is not organized the same way as in healthy individuals, causing an inability to keep a steady march; moreover, the level of akinesia changes according to the nigrostriatal dopamine decrease. The reason for muscle the relation between muscle rigidity and bradykinesia is not scientifically elucidated, but we know that it consists of a resistance increase throughout the amplitude of the passive movement [66,68]. However, there are reports that PD patients perform the activation of wrist and elbow agonist muscles at the same time that tremor activates, thus reducing the speed of movements [64,69].

Through functional magnetic resonance imaging (fMRI) studies, cortical changes induced by sequential and complex motor tasks were examined in both a control group and a group of akinetic parkinsonians. It was observed that hypoactivity occurred in supplementary motor and dorsolateral areas, inducing akinesia, and hyperactivity occurred in the caudal supplementary motor area, anterior cingulated and lateral premotor areas, sensorimotor activity and parietal cortex [70]. In particular, in monkeys with PD, we find a subcortical negligence, which seems to be consistent with the role of dopaminergic nigrostriatal neurons in regulating movement [71]. Thus, researches for understanding motion changes in individuals with PD have been widely studied. In general, when investigating the difficulties of coordination and visual feedback it was observed that individuals with PD significantly prolonged the time of movement; when the object was not visible, the time to reach the target increased. This suggests that individuals with PD have significant limitations in the adjustment of neural networks when they need to use internal representations, whether to catch an object or to see their hand movement [72]. Furthermore, when an individual with PD performs the task of grasping and lifting objects, the person initially shows deficiencies in strength mechanisms and finger amplitude, then recovered during movement [73].

A study with 14 PD patients was conducted in order to assess the effect of hearing and visual cues on gait initiation, using a rhythmic beep with time intervals and transverse lines on the ground with the maximum length of a step. It was observed that, after calculating the time and distance of the patient’s step through kinematic recordings, visual signals promoted better gait activity than auditory stimuli [74]. Additionally, another study examined the association between the efficiency of visuoperceptual and conceptual processing, measured by repetition priming, and by rate of change of Parkinsonian signs. It was observed that elderly subjects with better visuoperceptual conditions progressed more slowly during a follow-up (i.e., up to 11 years) towards the development of PD. The results also showed that individual differences in visuoperceptual processing efficiency occurred in older people without cognitive impairment, and promoted important changes in motor function. This may be an early sign of vulnerability in the corticostriatal circuit, which contributes to sensorimotor integration [75]. Thus, in patients with PD, the time response for motor initiation increases according to visuospatial difficulty [76,77].

### Time perception and sensorimotor integration

While studying PD it is essential to consider the contribution of sensorimotor integration, since this is responsible for the adjustment of the neural networks coordinating motor action [78]. Specifically, kinesthetic awareness, i.e. motion coordination, and proprioceptive information are considerably important for motor control [79,80], especially if the patient is less able to activate the muscle spindles, thus impairing sensorimotor integration [81,82].

Furthermore, some studies investigated the neural response deficits between the BG and both thalamus and supplementary motor area (SMA), which occur because of an unbalance between the direct and indirect circuits, thus increasing the quantity of the striatum stimuli going to the substantia nigra [80,83]. In another experiment using electrical stimulation, the authors analyzed finger movements in order to verify sensorimotor skills in PD patients. They observed that, whenever the BG generated an inappropriate motor command, this resulted in a slower movement performance and in a reduced spatial awareness [81].

Moreover, changes in postural control in individuals with PD are directly related to visuospatial perception, spatial attention and visuomotor control [84-86]. Therefore, a deficit in the process of sensorimotor integration may explain an impaired planning and preparation of motor commands in PD patients. For example, in blind individuals, the CNS seeks to reorganize and hierarchically prioritize sensory inputs as well as promote postural control adjustments to compensate for the visual deficiency. Nevertheless, in patients with PD, this compensatory mechanism is inefficient because the basal ganglia does not influence properly sensorimotor integration [81,87,88]; that is, conflicts in the basal ganglia prevent the final adjustment for appropriate posture control [89,90].

With regards to TP, the most accepted theory (i.e. Scalar Expectancy Theory) is that temporal cognitive information processing occurs in three different stages. First, during the timing phase, an intrinsic pacemaker sends pulses to an accumulator, which counts them. In the second stage, the information collected by the accumulator is sent to the temporary working memory, and finally, in the decision phase the current time is compared with time previously stored in memory to promote an appropriate time to response [91,92].

Other factors such as alertness, attention and intention to perform an action may alter and determine the individual’s perception of time. Attention affects the temporal processing of visual stimuli, accelerating them [93-95], while changes in the timing or memory phase affect the perception of duration and the estimation of the event duration [96]. Thus, in individuals with PD, the temporal deficits seem to be present mainly in perception and temporal processing [5,22]. For example, in a study by Lu et al. [97], a procedure was performed to understand the role of attention in PD. They observed that patients could not properly grasp common objects when receiving visual distracting stimuli, and this may be related to a deficient perception and time processing.

The temporal forecast is a mechanism that allows the individual to use sensory stimuli or motor acts to plan and achieve goals. This control may occur by means of endogenous and exogenous stimuli, which improve the accuracy and speed of the individual [36,98,99]. In this context, PD patients produce a time estimate shorter than healthy individuals. This was observed in the study by Pouthas and Perbal [96], who analyzed patients during a reading task. The results showed that a deficit in dopamine caused a delay in the internal clock and an underestimation of time.

Dusek et al. [100] examined the precuneus of twelve PD patients through functional magnetic resonance imaging (fRMI) while performing a task that consisted of two phases: encoding and reproducing time. The results showed a tendency to overestimate or underestimate intervals, i.e. differences in precuneus activation may be related to some aspects of time perception deficits; this suggests that dopamine may allow compensatory activation of the precuneus and consequently improve the time estimation. In addition to this, Geiser and Kaelin-Lang [101] analyzed the perception of auditory pulses between two groups of subjects with early PD (with and without administration of L-DOPA) and a healthy control group; they found that the group without administration of L-DOPA performed the task as well as the control group. However, the L-DOPA medicated group showed a significantly faster pulse perception. These findings are not in agreement with the study by Harrington et al. [102], probably due to different purposes and methodological aspects. In this case, the authors investigated the neural sources and their treatment response in a task involving TP. The findings indicated that impaired TP in PD was associated with nigrostriatal and mesocortical dysfunction in systems that modulate temporal and non-temporal control (See Table 1).

**Table 1:** Summary of studies investigating the impact of time perception on sensorimotor integration in PD

**Author**	**n**	**Methods**	**Protocol**	**Results**
**Sabatini et al. **[70]	12	fMRI	Sequential movement with the right hand; induced a clear activation in areas involved in motor execution and programming.	Subcortical putaminal dopamine deficit modifies cortical motor pathways in Parkinson and induces a underactivation of the rostral SMA and dorsolateral prefrontal cortex.
**Nenadic et al. **[5]	15	fMRI	Auditory time estimation task and frequency discrimination as an active control task.	Central processing of temporal information associated with basal ganglia activity. Temporal information processing in the brain might thus be a distributed process of interaction between modality-dependent sensory cortical function and attention and memory.
**Pastor et al. **[103]	14	fMRI	Simple detect the stimulus (control task) or to identify a stimulus property.	Pre-SMA and anterior cingulate were activated. Fronto-medial structures, directly and broadly connected with sensory and motor cortical areas of both hemispheres, as well as subcortical regions.
**Jantzen et al. **[104]	14	fMRI	Four conditions: synchronized pacing, synchronized continuation, syncopated pacing, and syncopated continuation.	Neural activity underlying continuation does not generalize across all timing contexts but is strongly influenced by the prior pacing context.
**Pouthas and Perbal.**[96]	46	Temporal accuracy	Temporal tasks, reproduction and production of duration in two conditions: control counting and concurrent reading.	The relationship between time estimation and cognitive functions – processing speed and memory – depend on the context in which duration is evaluated.
**Praamtra et al. **[99]	10	EEG	Choice reaction task with two different, temporally regular stimulus presentation regimes.	Interval timing for different behaviors relies on qualitatively similar mechanisms implemented in distinct cortical substrates.
**Wittmann et al. **[105]	13	fMRI	Six blocks of eight preference judgment trials each. On each trial, subjects were presented with a choice between a future and an immediate reward.	The striatum is critical for processing the magnitude of an option’s value over time. Inferior frontal activation pattern was associated with more consistent temporal discounting performance over time.
**Lee et al. **[45]	21	EEG	Subjects performed a visuospatial memory task.	Patients with Parkinson’s disease showed impairment at filtering out distracters, and they were able to hold fewer items in memory than control subjects. This indicates that the basal ganglia help controlling access to working memory.
**Grahn and Brett **[106]	30	NART and fMRI.	Subjects were tested on a discrimination paradigm.	Parkinson patients are specifically less able in processing beat-based sequences compared to non-beat-based ones. The basal ganglia and the medial pre-motor system therefore appear to be necessary for processing rhythms.

Legend: EEG = electroencephalography; fMRI = functional magnetic resonance imaging; MRI = magnetic resonance imaging; NART = National Adult Reading Test.

## Conclusion

Time perception is fundamental for the relationship between humans and their environment. Indeed, difficulties in “how to see the world” may characterize certain neurobehavioral clinical conditions such as PD [107]. This pathophysiological distortion may be improved starting from an analysis of the neurobiological model of interval timing, expressed within the conceptual framework of a traditional information-processing model called “Scalar Expectancy Theory”. Furthermore, the process of sensorimotor integration in the posterior parietal cortex is impaired due to a basal ganglia dysfunction, which provokes inadequate sensory stimuli inputs [107]. The growing interest in time perception and in its modification in PD will undoubtedly speed up our understanding of time-related disorders. In addition to possible pharmacological manipulations designed to affect the clock speed, the advancement and implementation of an external therapeutic/educational support facilitating the temporal dimensions of motor behavior may be particularly effective in promoting specific rehabilitation programs.

## Competing interests

The authors declared that they have no competing interests.

## Authors’ contributions

ML, FC, ST, PR and OAC participated in the definition of the study design and the protocol. Authors ML, FC, ST, AEN, SM, PR, and OAC managed the literature searches. Authors ML, FC, ST, SM, PR and OAC wrote the first draft of the manuscript. All authors contributed to and have approved the final manuscript.
